# Isochlorogenic acid C prevents enterovirus 71 infection via modulating redox homeostasis of glutathione

**DOI:** 10.1038/s41598-017-16446-7

**Published:** 2017-11-24

**Authors:** Zeyu Cao, Yue Ding, Liang Cao, Gang Ding, Zhenzhong Wang, Wei Xiao

**Affiliations:** State Key Laboratory of New-tech for Chinese Medicine Pharmaceutical Process, Jiangsu Kanion Pharmaceutical Co., Ltd, Lianyungang, 222001 China

## Abstract

Enterovirus 71 (EV71) is a key pathogen of hand, foot and mouth disease (HFMD) in children under 6 years of age. The antiviral potency of antioxidant isochlorogenic acid C (ICAC) extracted from foods was evaluated in cellular and animal models. First, the cytotoxicity of ICAC on Vero cells was investigated. The viral plaques, cytopathic effects and yield induced by EV71 infection were obviously reduced by ICAC, which was consistent with the investigation of *VP1* transcripts and protein expression. Moreover, the mortality, weight loss and limb paralysis of mice caused by EV71 challenge were remarkably relieved by ICAC injection, which was achieved through decreases in the viral load and cytokine secretion in the mouse brain. Further biochemical assays showed that ICAC modulated several antioxidant enzymes involved in reduced and oxidized glutathione (GSH and GSSG) homeostasis, including glutathione reductase (GR), glutathione peroxidase (GPX), and glucose-6-phosphate dehydrogenase (G6PD), resulting in restoration of the GSH/GSSG ratio and reactive oxygen species (ROS) level. Finally, the antiviral effects of ICAC were dose-dependently disrupted by BSO, a biosynthesis inhibitor of GSH. This study indicated that ICAC acted as an antioxidant and prevented EV71 infection by modulating the redox homeostasis of glutathione.

## Introduction

Human enterovirus 71 (EV71), which belongs to the genus *Enterovirus*, has been confirmed as a critical pathogen of hand, foot and mouth disease^1^. EV71 infection can cause severe symptoms, such as pulmonary oedema and brainstem and cerebellar encephalitis, which can lead to respiratory failure and death^2–5^. Cytokines in tissues, such as tumour necrosis factor-α (TNF-α), interleukin-6 (IL-6), and monocyte chemotactic protein-1 (MCP-1), can be induced by an excess viral load, resulting in tissue damage and chronic inflammation^6^, which play key roles in the pathogenicity and severity of EV71 infection^7–11^. In 2015, a vaccine against EV71 was approved as a new tool to control hand, foot and mouth disease (HFMD) outbreaks^12^. However, no specific chemical drug targeting EV71 has been approved. Thus, antiviral compounds must be screened for new drug development, and the mechanisms should be carefully discussed.

Viral infection can lead to oxidative stress^13^. A previous study showed that the redox status modulated host cell susceptibility to viruses^14^. For example, a shift in the intracellular redox milieu towards the oxidizing end enhanced viral replication and CPE^15^. Interestingly, the viral infection could be reversed by supplementing with glutathione (GSH), which indicated roles for GSH in antiviral defence^16^.

GSH plays a central role in reactive oxygen species (ROS) detoxification^17^. Briefly, glutathione peroxidase (GPX) catalyses the degradation of ROS coupled with the conversion of GSH to its oxidized form (GSSG). Glutathione reductase (GR) is an essential factor responsible for reducing GSSG back to GSH in the presence of NADPH, which is provided by glucose-6-phsophate dehydrogenase (G6PD)^17^. Upon viral infection, an imbalance in GSH redox homeostasis in host cells is observed^15^ due to the accumulation of ROS^18^, which can be reversed by exogenous supplementation of antioxidants^15,16^. L-buthionine sulfoximine (BSO) is a specific and selective inhibitor of γ- glutamylcysteine synthase and consequently of GSH synthesis^19^. Previous report^20^ showed BSO significantly reduced GSH level. Thus, BSO is usually used to deplete GSH in cells.

Isochlorogenic acid C (ICAC), which is a di-O-caffeoyl derivative of chlorogenic acid (CHA), is a well-known antioxidant^21–23^ from herbal plants^21^ that has revealed more potent effects than other isomers^24,25^. Previous reports showed that ICAC and its isomers exhibited a broad-spectrum antiviral potency against respiratory syncytial virus (RSV)^26^, human immunodeficiency virus (HIV)^27–29^, and coxsackievirus^30^. However, the potential effects of ICAC against EV71 are unknown and should be investigated. Only CHA (the parent nucleus of ICAC) has been reported to inhibit EV71 replication *in vitro* ^31^. However, ICAC extracted from *Flos Lonicerae* was indicated to reverse acetaminophen-induced liver injury via modulating the GSH content^32^. Thus, the potential regulation of GSH metabolism should be discussed with a focus on ICAC-derived inhibition of EV71 infection.

In this study, the antiviral efficacy of ICAC against EV71 was confirmed in both cellular and animal models. The compound was shown to reduce the mortality of mice upon EV71 challenge by decreasing the viral load and cytokine secretion. Further biochemical assays suggested that ICAC restored the GSH/GSSG ratio by regulating the enzymes responsible for GSH metabolism, resulting in a decreased ROS level. Taken together, the data in this study indicated that ICAC acted as an antioxidant and prevented EV71 infection via modulating GSH redox homeostasis.

## Results

### Cytotoxicity of ICAC

**Figure 1:**
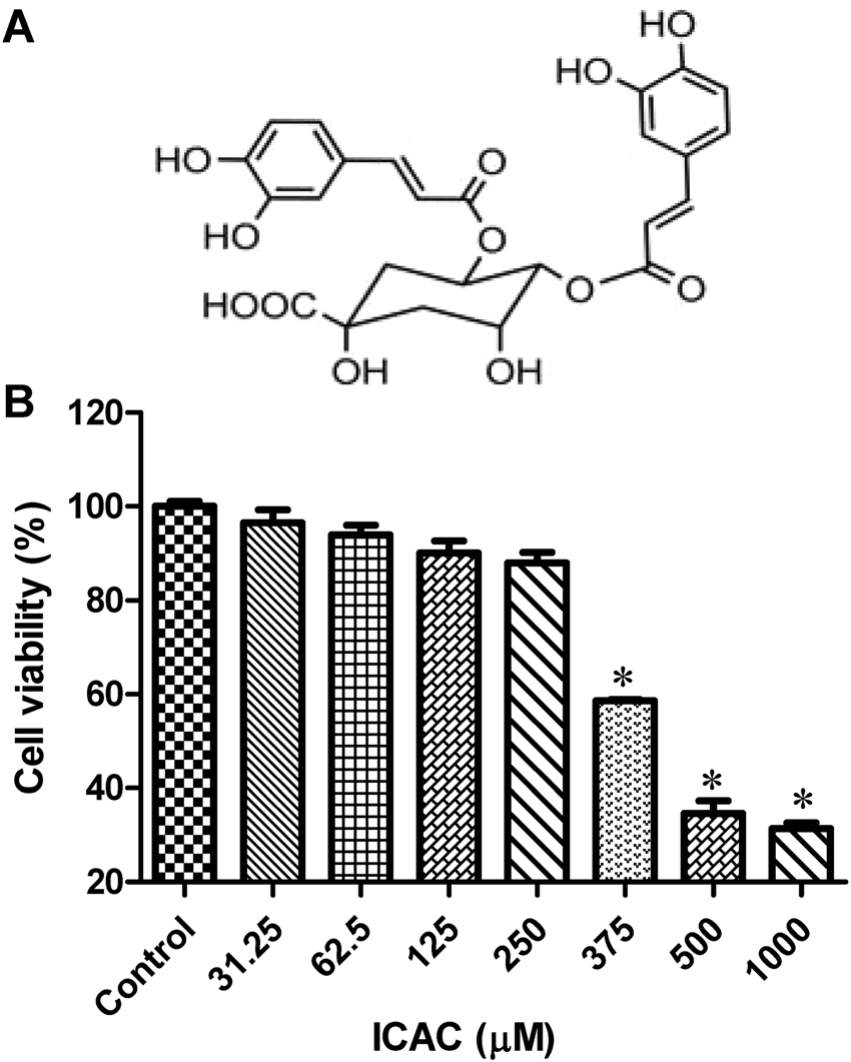
The molecular structure of ICAC and its effects on Vero cell viability. (**A**) The molecular structure of ICAC. (**B**) The compound was diluted to various concentrations as indicated in triplicate. The cytotoxicity of ICAC was determined by MTS assay after incubation with the drug for 48 h. The cell viability in DMEM without ICAC (control) was set as 100% (*n* = 6). All results were expressed as the means ± SEs. Asterisks indicate that the data significantly differ from the control group at the *P* < 0.05 level according to one-way analysis of variance.

The molecular structure of ICAC, which is also called 4,5-O-dicaffeoylquinic acid, is displayed in Fig. 1A. To investigate the toxicity of ICAC on Vero cells, the cell viability was determined after ICAC supplementation for 48 h. The compound exhibited minor cytotoxicity at concentrations up to 250 µM (Fig. 1B, *P* > 0.05). However, the cell viability decreased gradually and clearly when the concentration was increased to 375 µM or more in comparison with the viability of the blank control (Fig. 1B, *P* < 0.05). According to the results, the median toxic concentration (TC_50_) of ICAC for the cells was approximately 429 µM.

### Antiviral effects of ICAC against EV71 *in vitro*

**Figure 2:**
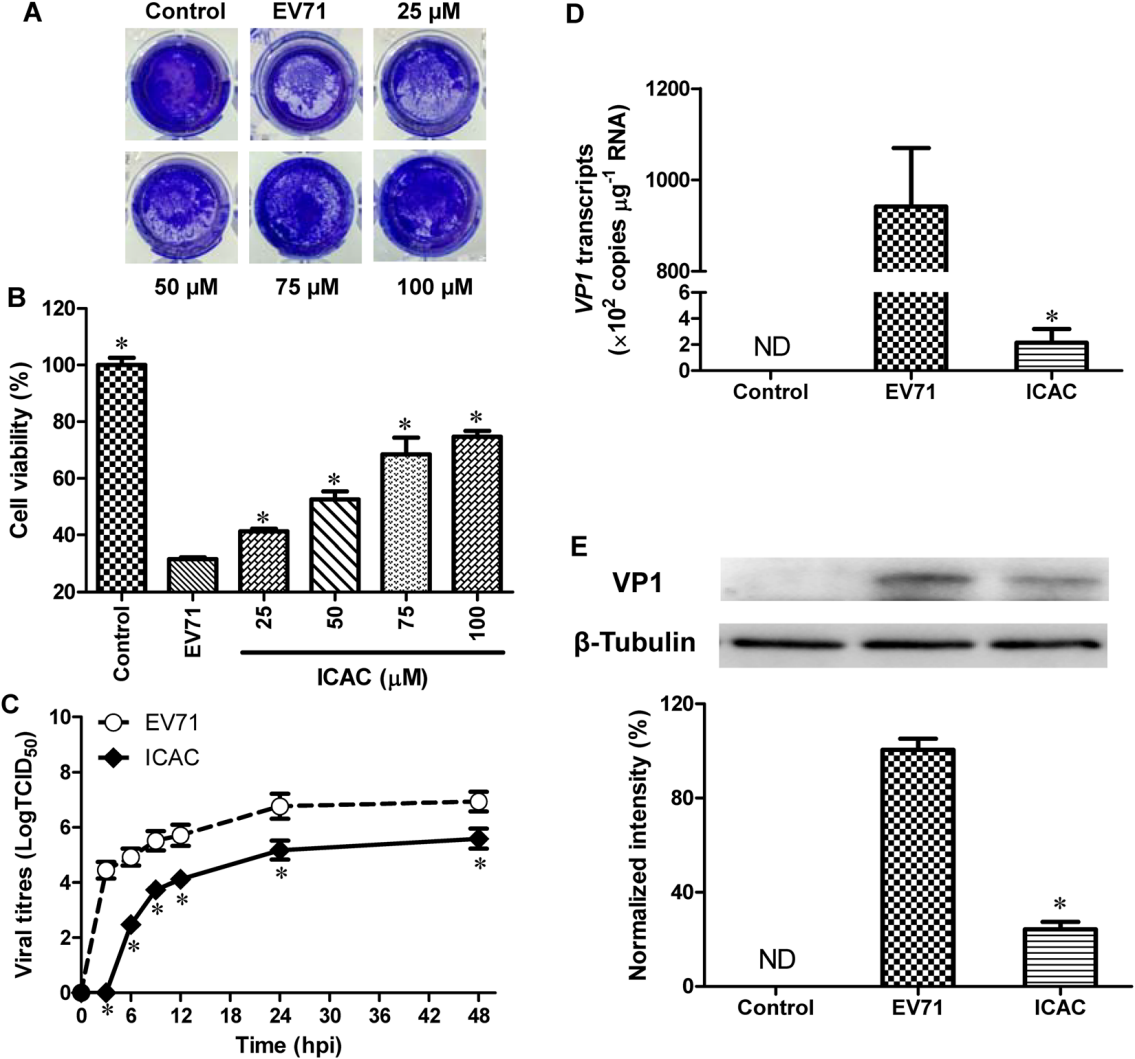
The antiviral effects of IACA against EV71 in Vero cells. Vero cells were infected with 100 TCID_50_ of EV71 with or without different ICAC concentrations as indicated. Uninfected cells were used as the control group. (**A**) ICAC blocked the CPE of EV71 infection. (**B**) Cell viability was detected using the MTS cell proliferation assay kit at 48 hpi. The viability of the control group was set as 100% (*n* = 6). (**C**) The virions were collected by freeze-thawing at the indicated time points. The supernatant was harvested for the viral titre assay. (**D**) The RNA load was determined using a real-time PCR kit specific for the *VP1* gene. (**E**) Protein samples normalized to 40 µg were subjected to 12.5% SDS-PAGE and then transferred to PVDF membrane to detect the EV71 VP1 protein expression levels. The amount of β-Tubulin was used as the internal standard. The VP1 band intensity was analysed and normalized to the corresponding band intensity of β-Tubulin. The band intensity of the EV71 group was set as 100% (*n* = 3). All results were expressed as the means ± SEs. Asterisks indicate that the data significantly differ from the EV71 group at the *P* < 0.05 level according to one-way analysis of variance.

The antiviral potency of ICAC was estimated in a cellular model. As expected, the plaques and CPE caused by EV71 infection were significantly reduced by ICAC in a dose-dependent manner (Fig. 2A,B). In particular, 100 µM ICAC, which had an inhibitory rate of 63.1%, clearly relieved the CPE caused by viral infection (Fig. 2B, *P* < 0.05). This concentration was appropriate for the inhibition of EV71 infection at a dose of 100 TCID_50_ and was utilized in the subsequent experiments. According to the results, the concentration required to obtain the 50% of maximal effect (EC_50_) and the selection index (SI = TC_50_/EC_50_) were approximately 72 µM and 5.92, respectively.

To investigate inhibition of the viral yield by ICAC, a time-course experiment was performed. As shown in Fig. 2C, EV71 replicated rapidly from 0–12 hours post-infection (hpi), followed by a gradual titre increase from 12–24 hpi that was delayed by ICAC addition. For instance, ICAC reduced the viral titres by approximately 39-fold at 12 hpi (Fig. 2C, *P* < 0.05) compared with the titres obtained by EV71 infection alone. Furthermore, the *VP1* mRNA transcript and protein levels were analysed. The *VP1* transcripts induced by infection were significantly restrained by ICAC administration at 12 hpi (Fig. 2D, *P* < 0.05). Western blotting analysis indicated that ICAC decreased the VP1 protein expression enhanced by EV71 infection at 12 hpi (Fig. 2E, *P* < 0.05). Taken together, our data demonstrated the antiviral effects of ICAC against EV71 in the Vero cell model.

### ICAC reduced the mortality of mice upon EV71 challenge

**Figure 3:**
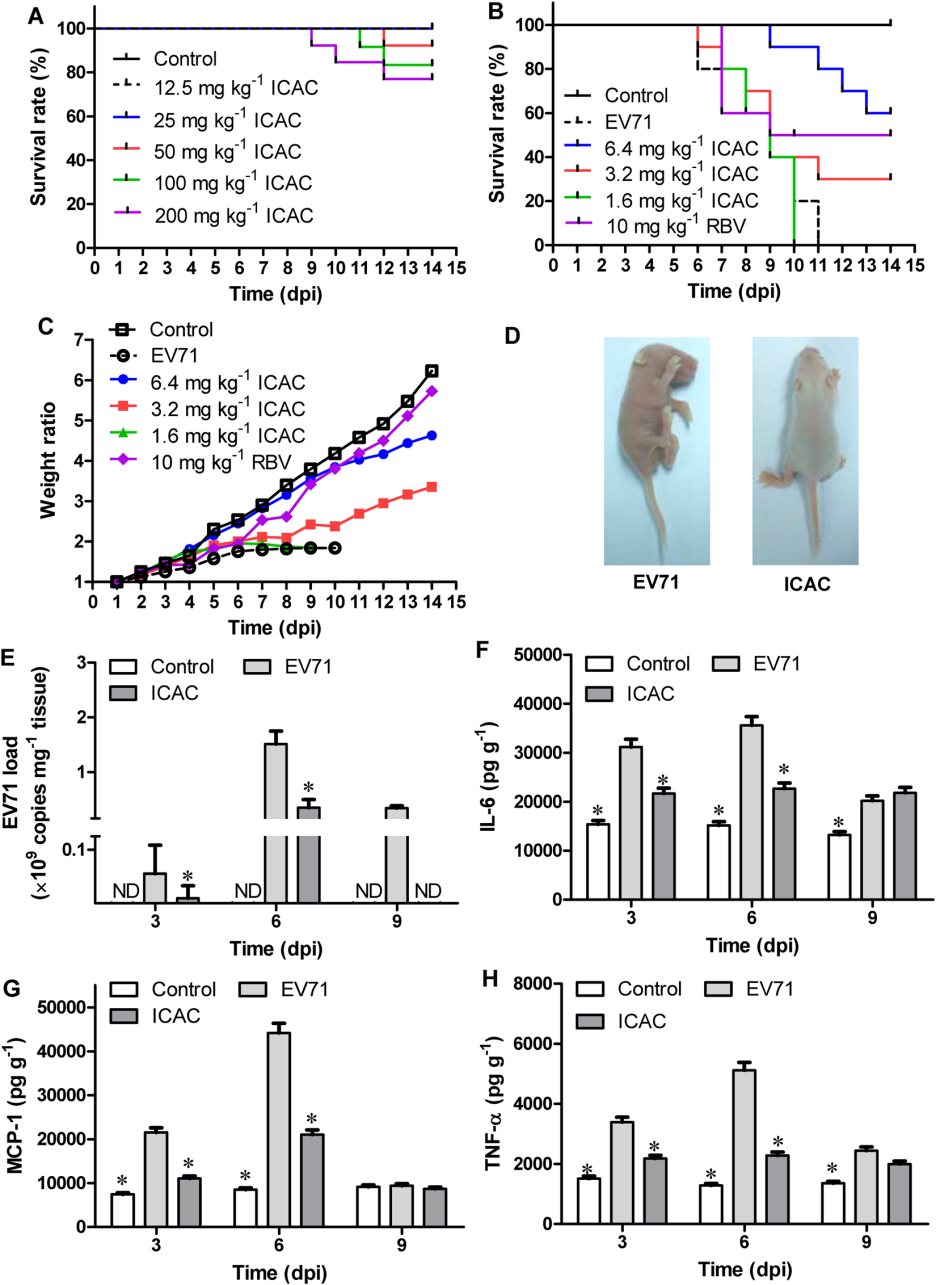
ICAC reduced the mortality of the EV71-infected mice by regulating the viral load and cytokine secretion. Two-day-old mice were utilized as the animal model. (**A**) The acute toxicity of ICAC (12.5–200 mg/kg) on mice was evaluated. In the antiviral study, the mice were challenged intraperitoneally with saline (control) or 1×10^7^ TCID_50_ of EV71. Then, the infected mice were treated with various doses (1.6–6.4 mg/kg) of ICAC or 10 mg/kg of ribavirin for 14 days. The mice in the model group were injected with saline. The survival rates (**B**) and body weight ratios (**C**) were recorded continuously until 14 dpi. The body weight ratio was presented relative to the corresponding treatments on the first day (*n* = 10). (**D**) Representative photos of limb paralysis caused by EV71 infection (left) and healthy phenotypes of mice treated with 6.4 mg/kg of ICAC (right) at 6 dpi. The brain tissues were sampled at 3, 6, and 9 dpi. (**E**) The viral loads in the brains were determined by real-time PCR (*n* = 6). The IL-6 (**F**), MCP-1 (**G**), and TNF-α (**H**) levels in the brains were determined by ELISA (*n* = 6). All results were expressed as the means ± SEs. Asterisks indicate that the data significantly differ from the EV71 group at the *P* < 0.05 level according to one-way analysis of variance.

To further confirm the inhibitory effects of ICAC against EV71, a suckling mouse model was utilized. First, the acute toxicity of ICAC was studied. ICAC at doses of less than 25 mg/kg daily for 14 days failed to cause any death or abnormal signs in the mice (Fig. 3A). Thus, the doses used in the subsequent animal experiments were nontoxic to the suckling mice. In the subsequent antiviral study, the model group mice revealed weight loss, paralysis, and mortality from 4–8 days post-infection (dpi), and all the mice died within 11 dpi (Table 1 and Fig. 3B–D). In contrast, ribavirin (10 mg/kg), which was used as a positive control drug, increased the survival rate to 50% and the survival time to 10.1 ± 1.8 days (Table 1 and Fig. 3B, *P* < 0.05). The weight loss caused by EV71 was also clearly alleviated by ribavirin (Fig. 3C). Moreover, administration of ICAC at a dose of 6.4 mg/kg obviously prevented the EV71-induced death by 60% and raised the survival time to 12.6 ± 0.6 days (Table 1 and Fig. 3B, *P* < 0.05). The symptoms, including growth inhibition (Fig. 3C), paralysis, and emaciation (Fig. 3D), caused by the viral infection were relieved by treatment with 6.4 mg/kg of ICAC. Additionally, 3.2 mg/kg of ICAC showed protective effects against mortality and growth inhibition in the infected mice (Table 1 and Fig. 3B,C), whereas 1.6 mg/kg of ICAC resulted in only weak effects on EV71 infection.

**Table 1:** ICAC reduced the mortality in EV71-infected mice.

Group	Total survival rate (%)	Survival period (Days)
Control	100^+^	14.0*
EV71	0	7.6 ± 0.9
6.4 mg kg^−1^ ICAC	60^+^	12.5 ± 0.6*
3.2 mg kg^−1^ ICAC	30^+^	9.4 ± 1.1*
1.6 mg kg^−1^ ICAC	0	8.0 ± 0.6
10 mg kg^−1^ RBV	50^+^	10.1 ± 1.8*

Each 2-day-old suckling mouse was intraperitoneally challenged with EV71 (1×10^7^ TCID_50_). Then, the infected mice were intraperitoneally injected with different daily doses of ICAC (1.6, 3.2, and 6.4 g kg^−1^) or 10 mg kg^−1^ of ribavirin in 0.1 mL saline for 14 days. The mice in the control (uninfected) were injected with the same volume of saline. Each group contained 10 suckling mice (*n* = 10). The survival rates of the mice were monitored daily. All results were expressed as the means ± SEs. Crosses and asterisks indicate that the data significantly differ from the EV71 group at the *P* < 0.05 level according to the Chi-square test and one-way analysis of variance, respectively.

### ICAC regulated the viral load and cytokine secretion in EV71-infected mice

The effects of ICAC on the viral load were investigated in the subsequent experiment. As shown in Fig. 3E, the viral load in the mouse brain tissues gradually increased to a peak at 6 dpi, followed by a decline at 9 dpi, which was obviously inhibited by ICAC at the indicated time points (Fig. 3E, *P* < 0.05). For example, the viral copies in the ICAC-treated mice decreased by approximately 17-fold at 6 dpi (Fig. 3E, *P* < 0.05), which was consistent with the results observed in the cellular model (Fig. 2D).

To further explore the regulation of cytokine secretion by ICAC, the IL-6, MCP-1, and TNF-α levels in the mouse brains were assessed at 3, 6, and 9 dpi. EV71 challenge resulted in significant IL-6, MCP-1, and TNF-α accumulation in the brain tissues at 3 and 6 dpi (Fig. 3F–H, *P* < 0.05) compared with the cytokine accumulation in the control group. ICAC relieved the accumulation of IL-6, MCP-1, and TNF-α clearly at 3 and 6 dpi (Fig. 3F–H, *P* < 0.05). Additionally, MCP-1 secretion in the brain tissues declined to near normal levels in the presence or absence of ICAC administration at 9 dpi (Fig. 3G), but ICAC failed to decrease IL-6 and TNF-α secretion in response to EV71 infection at 9 dpi (Fig. 3F,H). Therefore, ICAC regulated the viral load and the secretion of cytokines, including IL-6, MCP-1, and TNF-α, in EV71-infected mouse brains, which confirmed the antiviral effects of ICAC.

### ICAC mitigated the oxidative damage caused by EV71 infection via glutathione metabolism

**Figure 4:**
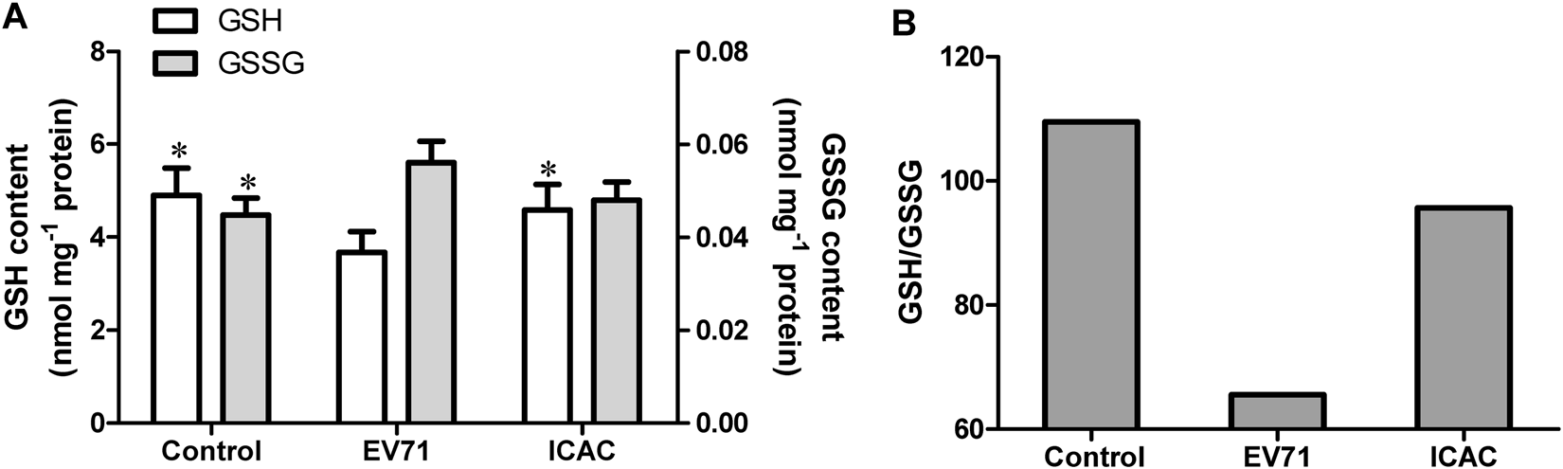
ICAC maintained GSH redox homeostasis in EV71-infected Vero cells. Infected (100 TCID_50_ of EV71) Vero cells were treated with medium or 100 µM ICAC for 12 h. Uninfected cells were used as the control group. The reduced and oxidized glutathione (GSH and GSSG) contents were analysed by HPLC (*n* = 3). All results were expressed as the means ± SEs. Asterisks indicate that the data significantly differ from the EV71 group at the *P* < 0.05 level according to one-way analysis of variance. The GSH/GSSG ratio was also calculated.

**Figure 5:**
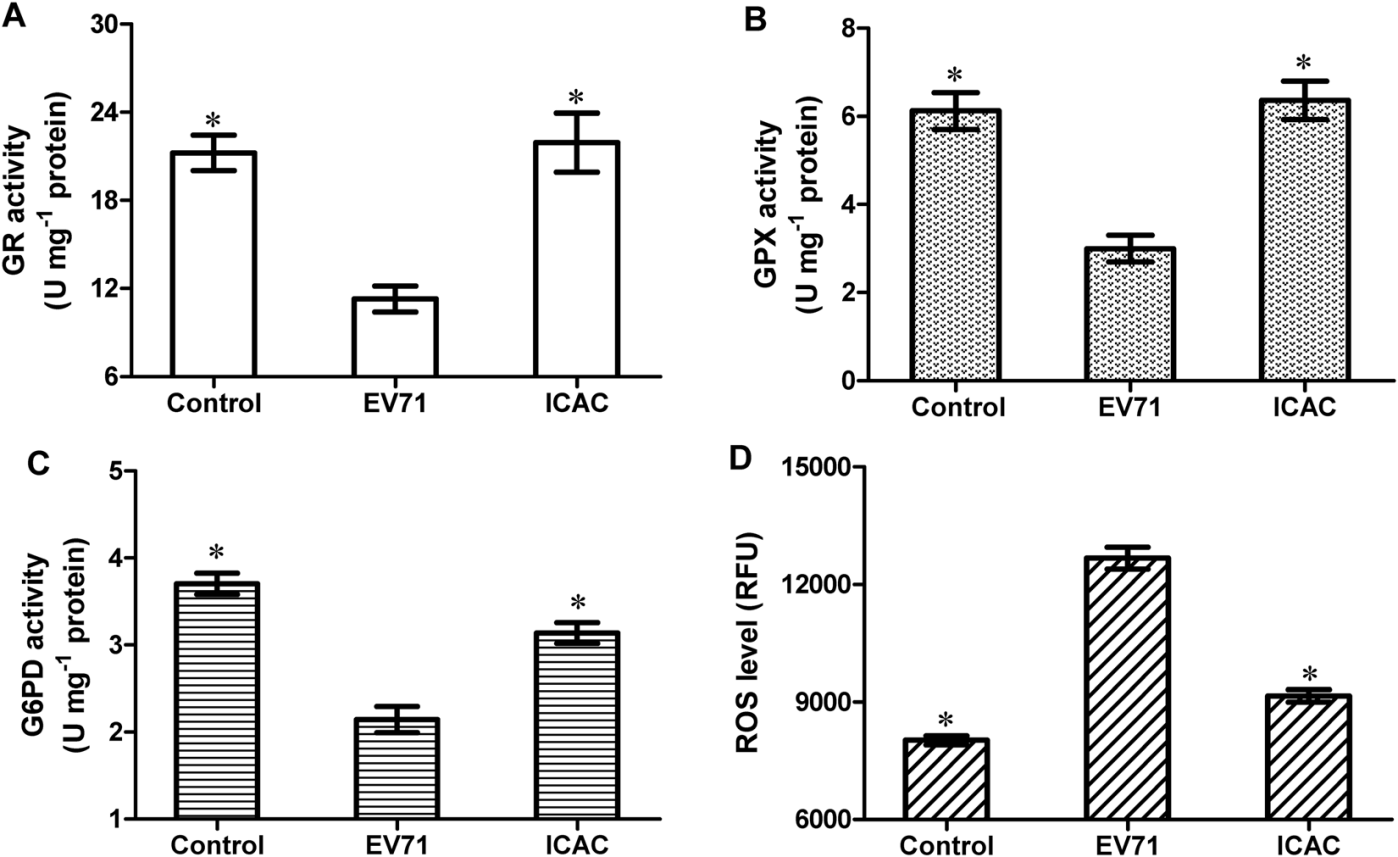
ICAC decreased the ROS level induced by EV71 infection via modulating the antioxidant enzymes involved in GSH metabolism. Infected (100 TCID_50_ EV71) Vero cells were treated with medium or 100 µM ICAC for 12 h. Uninfected cells were used as the control group. The antioxidant enzymes activities (**A**–**C**) and ROS levels (**D**) of the Vero cells were detected (*n* = 3). All results were expressed as the means ± SEs. Asterisks indicate that the data significantly differ from the EV71 group at the *P* < 0.05 level according to one-way analysis of variance.

To investigate the antiviral mechanism, the regulation of glutathione metabolism by ICAC was evaluated. Infection with 100 TCID_50_ of EV71 for 12 h induced a decrease in the GSH content that was coupled with an increase in GSSG in Vero cells (Fig. 4, *P* < 0.05). However, the ICAC treatment obviously reduced or eliminated the effects of EV71 infection on GSH and GSSG (Fig. 4, *P* < 0.05). Moreover, a quite high GSH/GSSG ratio, which is a key parameter for the intracellular redox status, was restored by ICAC addition compared with the ratio in the model group (Fig. 4B). Further analysis of GSH metabolic enzymes provided more insights into the exact mode of the antiviral action of ICAC against EV71. As shown, the activities of GR, GPX and G6PD in Vero cells were decreased by approximately 46.8%, 51.0% and 42.1%, respectively, following exposure to EV71 infection and were recovered by ICAC to varying degrees (Fig. 5). These results were consistent with the regulation of GSH redox homeostasis and the ROS level by ICAC (Fig. 4).

### BSO disrupted the antiviral effects of ICAC against EV71 in Vero cells

**Figure 6:**
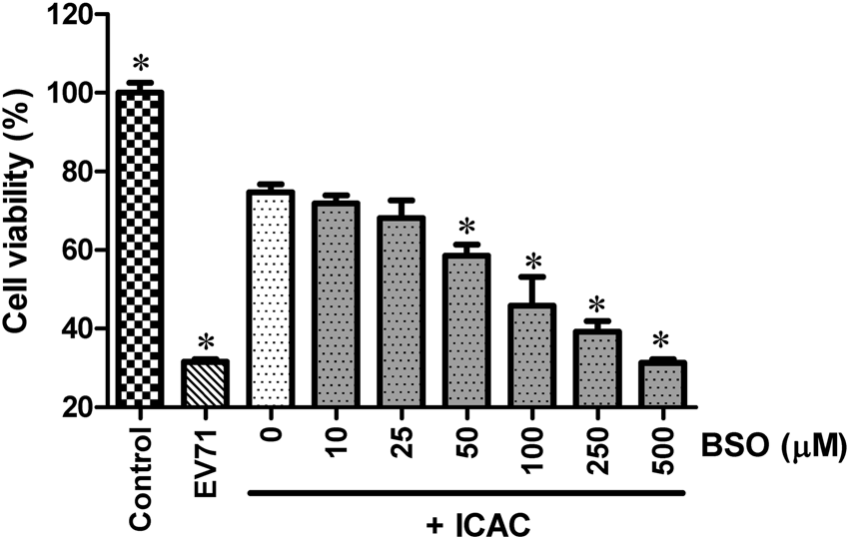
The antiviral effects of ICAC against EV71 were disrupted by BSO administration. Infected (100 TCID_50_ of EV71) Vero cells, treated with 100 µM ICAC, exposed to various concentrations of BSO (0~500 µM). Uninfected cells were used as the control group. Vero cells infected with 100 TCID_50_ of EV71 alone were used as the model group. Cell viability was detected using the MTS cell proliferation assay kit at 48 hpi. The viability of the control group was set as 100% (*n* = 6). Asterisks indicate that the data significantly differ from the ICAC group at the *P* < 0.05 level according to one-way analysis of variance.

To validate the potential effects of GSH induced by ICAC, BSO, a previously described inhibitor of GSH biosynthesis was utilized. As shown in Fig. 6, BSO disrupted the cellular defence effects of ICAC in a dose-dependent fusion. For example, when the concentration of BSO was increased to 500 µM, the protection of ICAC on Vero cells upon EV71 was abolished totally (Fig. 6). Meanwhile, results from preliminary experiment showed that BSO only revealed minor cytotoxicity on cellular viability at concentrations up to 2 mM (data not shown), which indicated the concentration used in our study was atoxic to the cells. On the other hand, it was also observed that exogenous GSH failed to inhibit the CPE caused by EV71 infection (data not shown). Thus, the data here exhibited GSH played a key role in ICAC-induced inhibition on EV71 infection, which confirmed the antiviral mechanism of ICAC.

## Discussion

ICAC is a natural product from *Lonicera japonica*, which is a well-known traditional Chinese herb that is widely used in HFMD treatments^33^. In addition to the capacity of ICAC to inhibit RSV^26^, HIV^27–29^, and coxsackievirus^30^ replication, we confirmed that the compound inhibited EV71 infection in cellular and animal models. In this study, ICAC revealed inhibitory potential against EV71 replication in Vero cells. In EV71-challenged mice, ICAC administration remarkably improved the survival rates and symptoms, including emaciation and paralysis. The compound also reduced the viral load and cytokine secretion in the brain. Finally, biochemical analysis showed that ICAC regulated the GSH/GSSG ratio and ROS level via the antioxidative enzymes involved in GSH metabolism. Taken together, the results demonstrated that ICAC inhibited EV71 infection via modulating GSH redox homeostasis in Vero cells.

In this study, the cytotoxicity of ICAC was investigated in the Vero cell model, which demonstrated an atoxic dose range. Vero cells, which are sensitive to EV71 infection^34–37^, were chosen as the cellular model to assess the antiviral effects of ICAC against the virus. Herein, the results indicated that ICAC reduced the plaques and CPE caused by EV71 infection in a dose-dependent manner. The time-dependent assay showed suppression of the EV71 yield by ICAC, which explained the protective effect of ICAC on cell viability. Moreover, *VP1* transcripts and protein synthesis, which are normally utilized for EV71 identification^38,39^, were decreased by ICAC treatment, which confirmed the inhibitory effect of ICAC on the EV71 yield. Taken together, these results demonstrate the antiviral capacity of ICAC against EV71. Previous reports have indicated that ICAC exhibits a broad antiviral spectrum^26–30,40–42^, and this study enriches the antiviral spectrum of ICAC. Our results indicate that ICAC may be the active ingredient that inhibits enteroviruses in the aqueous extract of *Lonicera japonica* ^30^ and the Reduning injection^43^. Moreover, ICAC exhibits potential utility in the control of HFMD via the necessary structural modification^44^.

The suckling mice challenged with EV71 gradually developed HFDM symptoms, including weight loss, paralysis, and death^45–47^. In this study, the mortality, weight loss, and limb paralysis caused by EV71 infection were alleviated significantly by ICAC administration. ICAC showed better effects than ribavirin injection, which is a clinical drug used for HFMD treatment^48^. Furthermore, ICAC decreased the viral load in the mouse brain^49^, which was confirmed by the results obtained in the cellular model. Previous research reported that ICAC inhibited HIV replication by inhibiting integrase activity^28,29^. However, the target through which ICAC reduced the EV71 yield is still unclear. Macrophages are an important target cell and may even be effectors of the EV71 attack^6,50^. Excessive cytokines and chemokines, including IL-6, MCP-1 and TNF-α, are secreted by the cells in response to the accumulation of viral copies^6^. In the central nervous system, these factors were proven to be responsible for the pathogenesis of the severe brainstem encephalitis and pulmonary oedema caused by EV71 infection^9–11,51^. In the present study, the steep accumulation of IL-6, MCP-1 and TNF-α in the infected mouse brains^52,53^ was reduced by ICAC, which correlated with the protection against mortality. Thus, the results revealed that ICAC inhibited EV71-induced mouse deaths by relieving the excessive viral load and cytokine secretion. However, the potential regulatory mechanisms underlying the effects of ICAC on the viral load and cytokine secretion should be discussed further.

To examine the antiviral mechanism of ICAC, we investigated modulation of GSH and its metabolism enzymes by the compound. GSH, which scavenges ROS in response to viral infection^54^, is an ideal biochemical to protect cells against oxidative stress. Maintenance of GSH redox homeostasis was reported to ameliorate cellular susceptibility to EV71 infection^15^. In this study, the depletion of the GSH pool induced by EV71 infection^18^ was reversed by ICAC supplementation, which correlated with the protection of ICAC on the cells upon to EV71 infection. Further biochemical analysis showed that ICAC up-regulated the enzymatic activities of GR, GPX and G6PD, which were responsible for GSH redox homeostasis. Additionally, these enzymes were indicated to play critical roles in the antiviral process. For example, GR and GPX exhibited protective effects against the myocarditis caused by coxsackievirus B3^55,56^. The increase in enzymatic activities by antioxidant administration correlated with the improved tolerance against viral infection^57–59^. Moreover, the increased susceptibility to EV71 infection was reversed by both G6PD expression and exogenous NAC treatment (a well-known antioxidant) in G6PD-deficient cells, which ensured the anti-EV71 role of G6PD^15^. The up-regulation of the enzymes, responsible for GSH metabolism, not only alleviated the oxidative damage caused by viral infection, but also maintained the redox homeostasis of GSH in Vero cells infected by EV71. Finally, the inhibition effects of GSH induced by ICAC on EV71 infection was proven by BSO addition, which usually used for GSH-depletion. Normally, renal intracellular GSH is degraded by γ-glutamyl transpeptidase into dipeptides and amino acids, which are translocated into cells^60,61^. Those findings could explain the observation that exogenous supplementation of GSH failed to prevent cells from CPE caused by EV71 infection. The present results indicated that regulation of GSH redox homeostasis could be the potential antiviral mechanism of ICAC against EV71. However, GSH was reported to be a novel factor essential for coxsackievirus virion morphogenesis, which indicated that the function of GSH in viral infection should be limited to the maintenance of redox homeostasis^62,63^. By contrary, EV71 morphogenesis was not observed to rely on GSH^63^. Thus, the function and regulation of GSH in EV71 infection and defence may be complex.

In summary, in this study, we showed the antiviral effects of ICAC against EV71 by inhibiting the viral yield. The compound improved the mortality and symptoms caused by viral infection by decreasing the EV71 load and cytokine secretion in the brain. ICAC maintained the GSH/GSSG ratio and its metabolic enzymatic activities. The antiviral effects of ICAC was disrupted by BSO, a biosynthesis inhibitor of GSH. Taken together, these results suggested that ICAC prevented EV71 infection via modulating GSH redox homeostasis.

## Methods

### Chemicals

Isochlorogenic acid C (ICAC, C_25_H_24_O_12_; CAS No. 32451-88-0; MW 516.45) was purchased from the National Institutes for Food and Drug Control of China (Beijing, China). The MTS cell proliferation assay kit was purchased from Promega Biotech Co., Ltd. (Beijing, China). Glutathione, oxidized glutathione, and L-buthionine sulfoximine (BSO) were purchased from Sigma-Aldrich (St. Louis, MO, USA).

### Virus and cells

EV71-infected Vero cells were used as the infection model^36,37^. The virus strain was isolated and identified clinically (GenBank accession no. HQ882182)^40^. EV71 was propagated in Vero cells, and the titres were determined as previously described^64^. The Vero cells were cultured in Dulbecco’s modified Eagle’s medium (DMEM) supplemented with 10% foetal bovine serum (FBS, Gibco) at 37 °C in a humidified incubator with 5% CO_2_.

### Animals

The 2-day-old BALB/c mice (1.6–2.0 g, SPF class) used as the animal model^43–45^ were purchased from Shanghai JSJ Experimental Animal Co. Ltd. (Shanghai, China). The mice were housed in an IVC system (temperature: 23 ± 2 °C, humidity: 40–70%, mechanical air supply, light-dark cycle: 12 h/12 h, illumination: 300 lux) with plenty of food and water. All animal experiment protocols were approved by Jiangsu Laboratory Animal Association (Licence number: SYXK(Jiangsu)2010-0010), which were conducted in accordance with the “Guiding Opinions on PETA’s” promulgated by Ministry of Science and Technology of China in 2006.

### Cellular toxicity assay

Vero cells (1×10^4^ cells/well) were seeded into 96-well plates and supplemented with various concentrations of ICAC as indicated in triplicate. The cell viability was detected using the MTS cell proliferation assay kit at 48 hours after addition.

### Antiviral study

Vero cells (1×10^4^ cells/well) seeded into 96-well plates were infected with EV71 (100 TCID_50_) at 37 °C for 2 h. After removing the virus, the cells were treated with various concentrations of ICAC as indicated in triplicate for 48 h. The plaque reduction assay was performed as previously described^40,65^. The cytopathic effects (CPEs) caused by the viral infection were measured quantitatively using the MTS cell proliferation assay kit according to the user manual. The concentration required for the 50% of maximal effect (EC_50_) and selection index (SI, SI = TC_50_/EC_50_) were calculated as previously described^38^. For the biochemical and molecular biology investigations, infected Vero cells (1 × 10^6^ cells/well in a 6-well plate) were treated with 100 µM ICAC for 12 h and then lysed for extraction.

### Time-course analysis of the EV71 yield

This analysis was performed as previously described^9,66,67^. Briefly, Vero cells (1 × 10^6^ cells/well) seeded into 6-well plates were infected with EV71 (100 TCID_50_) at 37 °C for 2 h. After removing the virus, the infected cells were treated with 100 µM ICAC. The viral particles were collected by freeze-thawing at −80 °C at the indicated time points.

### Viral load detection

Total RNA was isolated from the samples using the TRIzol reagent (Invitrogen, USA), and cDNA was synthesized using random hexamers with a reverse transcript kit (TaKaRa, China) according to the user manuals. The cDNA was subjected to the EV71 RNA Detection Kit (Shanghai ZJ Bio-Tech Co., Ltd) specific for the *VP1* gene. Positive fragments adjusted to a series of concentrations were used as a standard curve.

### Western blotting analysis

The samples were treated with RIPA lysis buffer (Beyotime Institute of Biotechnology, China) and centrifuged at 12,000 × g for 15 min at 4 °C. The protein concentrations were determined with a BCA protein assay kit (Beyotime Institute of Biotechnology, China). Western blotting analysis was performed as previously reported^67^.

### Mouse protection assay

Suckling mice were intraperitoneally challenged with EV71 (1×10^7^ TCID_50_). Infected mice were intraperitoneally injected with ICAC (1.6, 3.2, and 6.4 g/kg, qd) for 14 days. Ribavirin injection (10 mg/kg, qd) was used as a positive control. The model group was injected with the same volume (0.1 mL) of saline. The survival rates and body weights of the mice were monitored daily. For RNA and cytokine extraction, the mice were treated as described above, and the brains were sampled at 3, 6, and 9 dpi.

### Cytokine quantification

The brain tissues (~150 mg) were homogenized in 1 mL of 50 mM Tris buffer (pH 8.0) containing 5 M guanidine-HCl. The homogenates were mixed on an orbital shaker at room temperature for 4 h. The samples were diluted with cold PBS with 1× protease inhibitor cocktail (Thermo Scientific, USA) and then centrifuged at 16,000 × *g* for 20 min at 4 °C. The supernatants were used for the enzyme-linked immunosorbent assay (ELISA). The IL-6, MCP-1 and TNF-α levels were measured using anti-mouse ELISA kits (eBioscience, San Diego, CA, USA) according to the manufacturer’s guidelines.

### GSH and GSSG analyses

The GSH and GSSG levels were analysed by high-performance liquid chromatography (HPLC) as previously reported^16^.

### Enzymatic activity assays

The cells were lysed on ice using the Membrane and Cytosol Protein Extraction Kit (Beyotime Institute of Biotechnology, China) for the biochemical assays. The GR activity was determined using the Glutathione Reductase Assay Kit (Abcam, UK) according to the user manual. One unit was defined as the amount of enzyme that catalysed the conversion of 1.0 μmol of GSSG into GSH and generated 1.0 μmol of 5-merapto-2-nitrobenzoic acid under the assay kit conditions per minute at 25 °C. The GPX activity was detected using the Glutathione Peroxidase Assay Kit (Abcam, UK) according to the user manual. One unit was defined as the amount of enzyme that caused the oxidation of 1.0 µmol of NADPH to NADP^+^ under the assay kit conditions per minute at 25 °C. The G6PD activity was estimated using the Glucose 6-Phosphate Dehydrogenase Kit (Abcam, UK) according to the user manual. One unit was defined as the amount of enzyme that catalysed the conversion of 1.0 μmol of glucose-6-phosphate into 6- phosphoglucono-δ-lactone and generated 1.0 μmol of NADH under the assay kit conditions per minute at 37 °C.

### Determination of ROS

Vero cells (1 × 10^6^ cells/well) seeded in 6-well plate were infected and treated as described above. The ROS level was detected using a Cellular Reactive Oxygen Species Detection Kit (Abcam, UK) according to user manual. The relative fluorescence units (RFU) was monitored at Ex/Em = 540/570 nm.

### Statistics analysis

All results were expressed as the means ± SEs. The statistical significances of differences in the mean values were assessed with one-way analysis of variance (asterisk) or Chi-square test (cross) at the *P* < 0.05 level, respectively.

## References

[1] Schmidt NJ, Lennette EH, Ho HH (1974). An apparently new enterovirus isolated from patients with disease of the central nervous system. J. Infect. Dis..

[2] Huang X (2015). Epidemiological and etiological characteristics of hand, foot, and mouth disease in Henan, China, 2008–2013. Sci. Rep..

[3] Mizuta K (2009). Cross-antigenicity among EV71 strains from different genogroups isolated in Yamagata, Japan, between 1990 and 2007. Vaccine.

[4] Wang Y (2015). Enterovirus 71 infection in children with hand, foot, and mouth disease in Shanghai, China: epidemiology, clinical feature and diagnosis. Virol. J..

[5] Yang F (2009). Enterovirus 71 outbreak in the People’s Republic of China in 2008. J. Clin. Microbiol..

[6] Gong X (2012). Excessive proinflammatory cytokine and chemokine responses of human monocyte-derived macrophages to enterovirus 71 infection. BMC Infect. Dis..

[7] Han J (2014). Serum cytokine profiles of children with human enterovirus 71-associated hand, foot, and mouth disease. J. Med. Virol..

[8] Lin TY (2002). Different proinflammatory reactions in fatal and non-fatal enterovirus 71 infections: implications for early recognition and therapy. Acta Paediatr..

[9] Wang SM (2003). Pathogenesis of enterovirus 71 brainstem encephalitis in pediatric patients: roles of cytokines and cellular immune activation in patients with pulmonary edema. J. Infect. Dis..

[10] Wang SM (2007). Cerebrospinal fluid cytokines in enterovirus 71 brain stem encephalitis and echovirus meningitis infections of varying severity. Clin. Microbiol. Infect..

[11] Wang SM (2008). Acute chemokine response in the blood and cerebrospinal fluid of children with enterovirus 71-associated brainstem encephalitis. J. Infect. Dis..

[12] Mao QY, Wang Y, Bian L, Xu M, Liang Z (2016). EV71 vaccine, a new tool to control outbreaks of hand, foot and mouth disease (HFMD). Expert Rev. Vaccines.

[13] Schwarz KB (1996). Oxidative stress during viral infection: a review. Free Radic. Biol. Med..

[14] Beck MA, Handy J, Levander OA (2000). The role of oxidative stress in viral infections. Ann. N. Y Acad. Sci..

[15] Ho HY (2008). Glucose-6-phosphate dehydrogenase deficiency enhances enterovirus 71 infection. J. Gen. Virol..

[16] Cai J (2003). Inhibition of influenza infection by glutathione. Free Radic. Biol. Med..

[17] Meister A, Anderson ME (1983). Glutathione. Annu. Rev. Biochem..

[18] Cheng ML, Weng SF, Kuo CH, Ho HY (2014). Enterovirus 71 induces mitochondrial reactive oxygen species generation that is required for efficient replication. PLOS One.

[19] Griffith OW, Meister A (1979). Potent and specific inhibition of glutathione synthesis by buthionine sulfoximine (*S*-*n*-butyl homocysteine sulfoximine). J. Biol. Chem..

[20] Smith AD, Dawson H (2006). Glutathione is required for efficient production of infectious picornavirus virions. Virology.

[21] Aniya Y (2005). Free radical scavenging and hepatoprotective actions of the medicinal herb, Crassocephalum crepidioides from the Okinawa Islands. Biol. Pharm. Bull..

[22] Hwang SH, Paek JH, Lim SS (2016). Simultaneous ultra performance liquid chromatography determination and antioxidant activity of linarin, luteolin, chlorogenic acid and apigenin in different parts of Compositae species. Molecules.

[23] Guo W (2015). Isolation of isochlorogenic acid isomers in flower buds of Lonicera japonica by high-speed counter-current chromatography and preparative high performance liquid chromatography. J. Chromatogr. B Analyt. Technol. Biomed. Life Sci..

[24] dos Santos MD (2010). Effects of caffeoylquinic acid derivatives and C-flavonoid from Lychnophora ericoides on *in vitro* inflammatory mediator production. Nat. Prod. Commun..

[25] Park KH (2009). The anti-oxidative and anti-inflammatory effects of caffeoyl derivatives from the roots of Aconitum koreanum R. Raymond. Biol. Pharm. Bull..

[26] Ooi LS, Wang H, He Z, Ooi VE (2006). Antiviral activities of purified compounds from Youngia japonica (L.) DC (Asteraceae, Compositae) (Ooi, L. S., Wang, H., He, Z. & Ooi, V. E. Antiviral activities of purified compounds from Youngia japonica (L.) DC (Asteraceae, Compositae). J. Ethnopharmacol..

[27] Heyman HM (2015). Identification of anti-HIV active dicaffeoylquinic- and tricaffeoylquinic acids in Helichrysum populifolium by NMR-based metabolomic guided fractionation. Fitoterapia.

[28] Robinson WE, Reinecke MG, Abdel-Malek S, Jia Q, Chow SA (1996). Inhibitors of HIV-1 replication [corrected; erratum to be published] that inhibit HIV integrase. Proc. Natl Acad. Sci. USA.

[29] Robinson WE (1996). Dicaffeoylquinic acid inhibitors of human immunodeficiency virus integrase: inhibition of the core catalytic domain of human immunodeficiency virus integrase. Mol. Pharmacol..

[30] Yu Y (2013). Homosecoiridoid alkaloids with amino acid units from the flower buds of Lonicera japonica. J. Nat. Prod..

[31] Li X (2013). Chlorogenic acid inhibits the replication and viability of enterovirus 71 *in vitro*. PLOS One.

[32] Jiang P, Sheng YC, Chen YH, Ji LL, Wang ZT (2014). Protection of Flos Lonicerae against acetaminophen-induced liver injury and its mechanism. Environ. Toxicol. Pharmacol..

[33] Chen X (2013). A laboratory evaluation of medicinal herbs used in china for the treatment of hand, foot, and mouth disease. Evid. Based Complement. Alternat. Med..

[34] Chang SC, Lin JY, Lo LY, Li ML, Shih SR (2004). Diverse apoptotic pathways in enterovirus 71-infected cells. J. Neurovirol..

[35] Xi X (2013). The interplays between autophagy and apoptosis induced by enterovirus 71. PLOS One.

[36] Wang J (2013). Glycyrrhizic acid as the antiviral component of glycyrrhiza uralensis Fisch. Against coxsackievirus A16 and enterovirus 71 of hand foot and mouth disease. J. Ethnopharmacol..

[37] Weng TY (2005). Lactoferrin inhibits enterovirus 71 infection by binding to VP1 protein and host cells. Antiviral Res..

[38] Liu J (2011). Lycorine reduces mortality of human enterovirus 71-infected mice by inhibiting virus replication. Virol. J..

[39] Oberste MS, Nix WA, Maher K, Pallansch MA (2003). Improved molecular identification of enteroviruses by RT-PCR and amplicon sequencing. J. Clin. Virol..

[40] Wang CY (2013). Eupafolin and ethyl acetate fraction of Kalanchoe gracilis stem extract show potent antiviral activities against enterovirus 71 and coxsackievirus A16. Evid. Based Complement. Alternat. Med..

[41] Wang GF (2009). Anti-hepatitis B virus activity of chlorogenic acid, quinic acid and caffeic acid *in vivo* and *in vitro*. Antiviral Res..

[42] Xie Y (2013). Caffeic acid derivatives: a new type of influenza neuraminidase inhibitors. Bioorg. Med. Chem. Lett..

[43] Cao ZY, Chang XJ, Zhao ZP, Cao L, Xiao W (2015). Antiviral effects of Reduning injection against enterovirus 71 and possible mechanisms of action. Chin. J. Nat. Med..

[44] Huang LH (2016). Pharmacokinetics of isochlorgenic acid C in rats by HPLC-MS: absolute bioavailability and dose proportionality. J. Ethnopharmacol..

[45] Li P (2016). Genome analysis of enterovirus 71 strains differing in mouse pathogenicity. Virus Genes.

[46] Li Z (2014). *In vivo* time-related evaluation of a therapeutic neutralization monoclonal antibody against lethal enterovirus 71 infection in a mouse model. PLOS One.

[47] Lin P, Gao L, Huang Y, Chen Q, Shen H (2015). An enterovirus 71 strain causes skeletal muscle damage in infected mice. Int. J. Clin. Exp. Pathol..

[48] Li ZH (2008). Ribavirin reduces mortality in enterovirus 71-infected mice by decreasing viral replication. J. Infect. Dis..

[49] Lee YRW, Wang PS, Wang JR, Liu HS (2014). Enterovirus 71-induced autophagy increases viral replication and pathogenesis in a suckling mouse model. J. Biomed. Sci..

[50] Wang SM (2010). Enterovirus 71 infection of monocytes with antibody-dependent enhancement. Clin. Vaccine Immunol..

[51] Husseini RH, Sweet C, Collie MH, Smith H (1982). Elevation of nasal viral levels by suppression of fever in ferrets infected with influenza viruses of differing virulence. J. Infect. Dis..

[52] Hsiao HB (2014). Toll-like receptor 9-mediated protection of enterovirus 71 infection in mice is due to the release of danger-associated molecular patterns. J. Virol..

[53] Lee YP, Wang YF, Wang JR, Huang SW, Yu CK (2012). Enterovirus 71 blocks selectively type I interferon production through the 3C viral protein in mice. J. Med. Virol..

[54] Giblin FJ (2000). Glutathione: a vital lens antioxidant. J. Ocul. Pharmacol. Ther..

[55] Beck MA, Esworthy RS, Ho YS, Chu FF (1998). Glutathione peroxidase protects mice from viral-induced myocarditis. FASEB J..

[56] Chen LY, Tian XL, Yang B (1990). A study on the inhibition of rat myocardium glutathione peroxidase and glutathione reductase by moniliformin. Mycopathologia.

[57] Beck MA, Williams-Toone D, Levander OA (2003). Coxsackievirus B3-resistant mice become susceptible in Se/vitamin E deficiency. Free Radic. Biol. Med..

[58] Sartori G (2016). Antiviral action of diphenyl diselenide on herpes simplex virus 2 infection in female BALB/c mice. J. Cell. Biochem..

[59] Smith AD, South PK, Levander OA (2001). Effect of gold(I) compounds on the virulence of an amyocarditic strain of coxsackievirus B3. Biol. Trace Elem. Res..

[60] Griffith OW, Meister A (1979). Glutathione: interorgan translocation, turnover, and metabolism. Proc. Natl. Acad. Sci. USA.

[61] Griffith OW, Meister A (1979). Translocation of intracellular glutathione to membrane-bound γ-glutamyl transpeptidase as a discrete step in the γ-glutamyl cycle: glutathionuria after inhibition of transpeptidase. Proc. Natl. Acad. Sci. USA.

[62] Ma HC (2014). An interaction between glutathione and the capsid is required for the morphogenesis of C-cluster enteroviruses. PLOS Pathog..

[63] Thibaut HJ (2014). Binding of glutathione to enterovirus capsids is essential for virion morphogenesis. PLOS Pathog..

[64] Reed LJM, Muench H (1938). A simple method of estimating fifty percent endpoints. Am. J. Hyg..

[65] Lin YJ (2013). Inhibition of enterovirus 71 infections and viral IRES activity by Fructus Gardeniae and geniposide. Eur. J. Med. Chem..

[66] Cao Z (2016). Luteoloside acts as 3C protease inhibitor of enterovirus 71 *in vitro*. PLOS One.

[67] Lu J (2011). Viral kinetics of enterovirus 71 in human abdomyosarcoma cells. World J. Gastroenterol..

